# Redo procedures after sinus node sparing hybrid ablation for inappropriate sinus tachycardia/postural orthostatic sinus tachycardia

**DOI:** 10.1093/europace/euad373

**Published:** 2023-12-29

**Authors:** Carlo de Asmundis, Lorenzo Marcon, Luigi Pannone, Domenico Giovanni Della Rocca, Dhanunjaya Lakkireddy, Thomas M Beaver, Chad R Brodt, Cinzia Monaco, Antonio Sorgente, Charles Audiat, Giampaolo Vetta, Robbert Ramak, Ingrid Overeinder, Rani Kronenberger, Gezim Bala, Alexandre Almorad, Erwin Ströker, Juan Sieira, Andrea Sarkozy, Pedro Brugada, Gian Battista Chierchia, Mark La Meir

**Affiliations:** Heart Rhythm Management Centre, Postgraduate Program in Cardiac Electrophysiology and Pacing, Universitair Ziekenhuis Brussel - Vrije Universiteit Brussel, European Reference Networks Guard-Heart, Laarbeeklaan 101, 1090 Brussels, Belgium; Heart Rhythm Management Centre, Postgraduate Program in Cardiac Electrophysiology and Pacing, Universitair Ziekenhuis Brussel - Vrije Universiteit Brussel, European Reference Networks Guard-Heart, Laarbeeklaan 101, 1090 Brussels, Belgium; Heart Rhythm Management Centre, Postgraduate Program in Cardiac Electrophysiology and Pacing, Universitair Ziekenhuis Brussel - Vrije Universiteit Brussel, European Reference Networks Guard-Heart, Laarbeeklaan 101, 1090 Brussels, Belgium; Heart Rhythm Management Centre, Postgraduate Program in Cardiac Electrophysiology and Pacing, Universitair Ziekenhuis Brussel - Vrije Universiteit Brussel, European Reference Networks Guard-Heart, Laarbeeklaan 101, 1090 Brussels, Belgium; Kansas City Heart Rhythm Institute, Overland Park, KS, USA; Division of Thoracic and Cardiovascular Surgery, Department of Surgery, University of Florida, Gainesville, FL, USA; Sequoia Hospital, Palo Alto, CA, USA; Heart Rhythm Management Centre, Postgraduate Program in Cardiac Electrophysiology and Pacing, Universitair Ziekenhuis Brussel - Vrije Universiteit Brussel, European Reference Networks Guard-Heart, Laarbeeklaan 101, 1090 Brussels, Belgium; Heart Rhythm Management Centre, Postgraduate Program in Cardiac Electrophysiology and Pacing, Universitair Ziekenhuis Brussel - Vrije Universiteit Brussel, European Reference Networks Guard-Heart, Laarbeeklaan 101, 1090 Brussels, Belgium; Heart Rhythm Management Centre, Postgraduate Program in Cardiac Electrophysiology and Pacing, Universitair Ziekenhuis Brussel - Vrije Universiteit Brussel, European Reference Networks Guard-Heart, Laarbeeklaan 101, 1090 Brussels, Belgium; Heart Rhythm Management Centre, Postgraduate Program in Cardiac Electrophysiology and Pacing, Universitair Ziekenhuis Brussel - Vrije Universiteit Brussel, European Reference Networks Guard-Heart, Laarbeeklaan 101, 1090 Brussels, Belgium; Heart Rhythm Management Centre, Postgraduate Program in Cardiac Electrophysiology and Pacing, Universitair Ziekenhuis Brussel - Vrije Universiteit Brussel, European Reference Networks Guard-Heart, Laarbeeklaan 101, 1090 Brussels, Belgium; Heart Rhythm Management Centre, Postgraduate Program in Cardiac Electrophysiology and Pacing, Universitair Ziekenhuis Brussel - Vrije Universiteit Brussel, European Reference Networks Guard-Heart, Laarbeeklaan 101, 1090 Brussels, Belgium; Cardiac Surgery Department, Universitair Ziekenhuis Brussel, Vrije Universiteit Brussel, Brussels, Belgium; Heart Rhythm Management Centre, Postgraduate Program in Cardiac Electrophysiology and Pacing, Universitair Ziekenhuis Brussel - Vrije Universiteit Brussel, European Reference Networks Guard-Heart, Laarbeeklaan 101, 1090 Brussels, Belgium; Heart Rhythm Management Centre, Postgraduate Program in Cardiac Electrophysiology and Pacing, Universitair Ziekenhuis Brussel - Vrije Universiteit Brussel, European Reference Networks Guard-Heart, Laarbeeklaan 101, 1090 Brussels, Belgium; Heart Rhythm Management Centre, Postgraduate Program in Cardiac Electrophysiology and Pacing, Universitair Ziekenhuis Brussel - Vrije Universiteit Brussel, European Reference Networks Guard-Heart, Laarbeeklaan 101, 1090 Brussels, Belgium; Heart Rhythm Management Centre, Postgraduate Program in Cardiac Electrophysiology and Pacing, Universitair Ziekenhuis Brussel - Vrije Universiteit Brussel, European Reference Networks Guard-Heart, Laarbeeklaan 101, 1090 Brussels, Belgium; Heart Rhythm Management Centre, Postgraduate Program in Cardiac Electrophysiology and Pacing, Universitair Ziekenhuis Brussel - Vrije Universiteit Brussel, European Reference Networks Guard-Heart, Laarbeeklaan 101, 1090 Brussels, Belgium; Heart Rhythm Management Centre, Postgraduate Program in Cardiac Electrophysiology and Pacing, Universitair Ziekenhuis Brussel - Vrije Universiteit Brussel, European Reference Networks Guard-Heart, Laarbeeklaan 101, 1090 Brussels, Belgium; Heart Rhythm Management Centre, Postgraduate Program in Cardiac Electrophysiology and Pacing, Universitair Ziekenhuis Brussel - Vrije Universiteit Brussel, European Reference Networks Guard-Heart, Laarbeeklaan 101, 1090 Brussels, Belgium; Cardiac Surgery Department, Universitair Ziekenhuis Brussel, Vrije Universiteit Brussel, Brussels, Belgium

**Keywords:** Sinus node, inappropriate sinus node tachycardia, postural orthostatic sinus tachycardia, hybrid ablation, redo ablation

## Abstract

**Aims:**

A novel sinus node (SN) sparing hybrid ablation for inappropriate sinus node tachycardia (IST)/postural orthostatic tachycardia syndrome (POTS) has been demonstrated to be an effective and safe therapeutic option in patients with symptomatic drug-resistant IST/POTS. The aim of this study was to evaluate the long-term rate of redo procedures after hybrid IST ablation and procedural strategy, outcomes and safety of redo procedures.

**Methods and results:**

All consecutive patients from 2015 to 2023 were prospectively enrolled in the UZ Brussel monocentric IST/POTS registry. They were analysed if the following inclusion criteria were fulfilled: 1) diagnosis of IST or POTS, 2) symptomatic IST/POTS refractory or intolerant to drugs, and 3) hybrid SN sparing ablation performed. The primary endpoint was redo procedure. The primary safety endpoint was pacemaker (PM) implantation. A total of 220 patients undergone to hybrid IST ablation were included, 185 patients (84.1%) were treated for IST and 61 patients (27.7%) for POTS.

After a follow-up of 73.3 ± 16.2 months, 34 patients (15.4%) underwent a redo. A total of 23 patients (67.6%) had a redo for IST recurrence and 11 patients (32.4%) for other arrhythmias. Pacemaker implantation was performed in 21 patients (9.5%). Nine patients (4.1%) had no redo procedure and experienced sick sinus syndrome requiring a PM. Twelve patients (5.4%) received a PM as a shared therapeutic choice combined with SN ablation procedure.

**Conclusion:**

In a large cohort of patients the long-term free survival from redo procedure after hybrid IST ablation was 84.6% with a low PM implantation rate.

## Graphical Abstract

**Graphical abstract:**
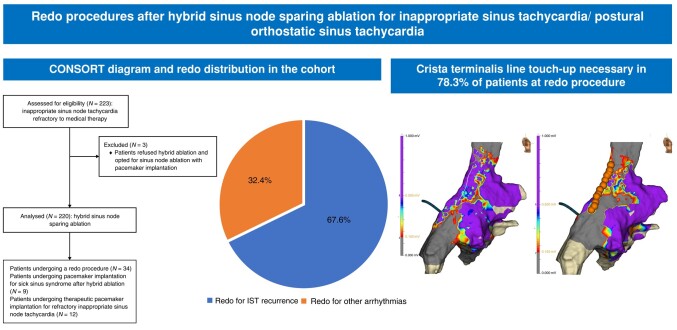
At long-term follow-up of ≍6 years, after hybrid inappropriate sinus node tachycardia (IST) ablation 15.4% of patients underwent to a redo procedure. A total 67.6% of patients had a redo procedure for IST recurrence and 32.4% of patients for other arrhythmias. In most patients undergoing a redo procedure a gap in at least one previous line, was found and the most common gap was in the crista terminalis line.

**What’s new?**

- In a cohort of 220 patients undergoing sinus node sparing hybrid inappropriate sinus node tachycardia ablation the long-term free survival from redo procedure was 84.6% with a low PM implantation rate.
- In most patients undergoing a redo procedure a gap in at least one previous line was found and the most common gap was in the crista terminalis line.

## Introduction

Inappropriate sinus node tachycardia (IST) is defined as a non-physiological elevation in resting heart rate. IST is a chronic condition in which the sinus rate exceeds physiological needs and cannot be unexplained by triggers or other medical conditions. Inappropriate sinus node tachycardia is defined as a sinus heart rate > 100 BPM at rest (with a mean 24 h heart rate > 90 BPM not due to primary causes) and is associated with distressing symptoms of palpitations.^1–4^

Symptoms are heterogeneous and include the following: palpitations, dyspnoea, exercise intolerance, syncope, fatigue, chest pain, anxiety, and depression.^3^

Inappropriate sinus node tachycardia is not rare, as its prevalence is up to is 1.2–5.0% in general population, and up to 90% of patients are young woman with an average age of 30 years.^5–8^

Postural orthostatic tachycardia syndrome (POTS) is a systemic illness, characterised by a variety of symptoms occurring with standing, such as palpitations, weakness, light-headedness, fainting, exercise intolerance, and fatigue. These symptoms are associated with an increase of >30 BPM (>40 BPM in patients 12–19 years of age) upon assuming an upright posture, without orthostatic hypotension (a drop of >20 mmHg in systolic blood pressure).^9,10^

Medical treatment of IST and POTS is suboptimal, with ≍30% of patients of patients not responding to β-blockers or ivabradine.^11^

A novel sinus node (SN) sparing hybrid ablation for IST/POTS has been demonstrated to be an effective and safe therapeutic option in patients with symptomatic drug-resistant IST and POTS. At long-term follow-up (≍4 years) after hybrid SN sparing ablation, 80% of patients showed a significant reduction in HR compared to the pre-ablation period.^9^ The efficiency of this strategy could be secondary to its potential targeting of increased automaticity (through local modulation of ganglia within the fat pad near the right pulmonary veins for POTS patients), SN re-entry (through the superior vena cava, inferior vena cava, and intercaval ablation lines), and, eventually, caval latent pacemakers or micro-reentry.^3^ In a multicentre prospective registry SN sparing hybrid ablation was associated with a 100% acute success rate and a rate of pacemaker (PM) implantation of 4%. A total of 8% of patients in this group underwent a subsequent redo procedure at 12 months follow-up.^12^

To date, no studies evaluated the clinical and procedural characteristics of patients undergoing to a redo procedure after hybrid SN sparing IST ablation.

The aims of this study were (1) to evaluate the long-term rate of redo procedures after hybrid IST ablation and (2) to evaluate procedural strategy, outcomes and safety of redo procedures after hybrid IST ablation.

## Methods

### Study design and patient population

All consecutive patients diagnosed with IST/POTS from June 2015 to May 2023 were prospectively enrolled in the UZ Brussel monocentric IST/POTS registry. They were analysed if the following inclusion criteria were fulfilled: 1) diagnosis of IST or POTS following current guidelines^4,13^, 2) symptomatic IST/POTS refractory or intolerant to drugs (e.g. β-blocker, calcium-channel blockers, ivabradine) and non-pharmacological therapy (e.g. physiotherapy, sleep hygiene and physical activity), and 3) hybrid SN sparing IST ablation performed following our described approach.^9,12,14^

**Figure 1:**
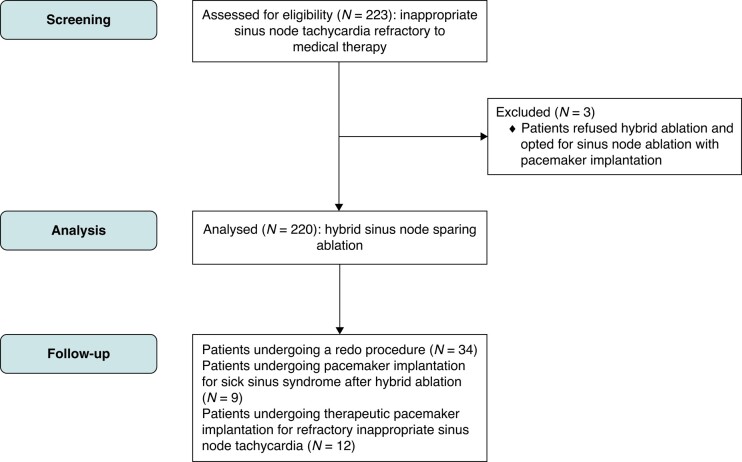
CONSORT diagram for patients screened and analysed in the study. CONSORT diagram for the patient cohort.

All patients were evaluated by a cardiologist together with a neurologist with both being experts in IST and POTS. Other causes of sinus tachycardia or any supraventricular tachycardia were ruled out with clinical, laboratory data and with an invasive electrophysiological study. All patients underwent a 12-lead ECG to confirm normal *P*-wave morphology and a Holter 24 h ECG to evaluate the mean heart rate and the circadian variation. Additional evaluations included blood testing (complete blood count, thyroid function, renal function and electrolytes, metabolic panel, drug testing and serum and urine catecholamines). Patients who proved refractory to or intolerant of pharmacologic treatment were offered hybrid SN sparing ablation treatment. Three patients refused hybrid ablation treatment and opted for conventional SN ablation and PM implantation. They were therefore excluded from the current study. The CONSORT diagram for patients enrolled in the study is summarised in *Figure 1*.

All patients signed an informed consent that had been approved by our institutional review board. The study complied with the Declaration of Helsinki as revised in 2013; the ethic committee approved the study.

### Hybrid ablation procedure

Hybrid SN sparing IST ablation approach has been previously described.^9,12,14^ Briefly, the right chest is accessed with three 5 mm working ports: a camera port is placed in the fifth intercostal space at the mid-axillary line. Two other working ports are added: (1) a 5 mm port for instruments in the third intercostal space at the anterior axillary line and (2) a 5 mm port for instruments in the seventh intercostal space at the anterior axillary line. After placement of the camera port, CO_2_ insufflation is used to increase the working space and to displace the diaphragm down. In women, the lateral mammary fold is usually used. The pericardium is opened with an endoscopic coagulation hook and/or scissors longitudinally, anterior to the phrenic nerve until visualisation of the superior cava vein (SVC) and inferior cava vein (IVC). The pericardial reflection of the IVC is then bluntly dissected until the oblique sinus. Endocardial mapping of the SN is performed, and the position of the endocardial catheter is observed using the thoracoscopic video system. Sinus node location is marked by the surgeon with methylene blue based on the position of the endocardial catheter (SN sparing approach). A bipolar bidirectional radiofrequency (RF) clamping device (EMR2, AtriCure Inc., Mason, OH, USA) is positioned over the SVC at the junction with the right atrium (RA) to isolate SVC. The same approach is performed to isolate IVC. To complete the hybrid IST ablation set, the crista terminalis line is performed with the clamp positioned in the oblique sinus and the anterior jaw over Waterston’s groove, covering the crista terminalis. Then, endocardial mapping confirms ablation line block. If lines are not blocked additional epicardial and/or endocardial ablations are performed. The pericardium is closed, and the right lung inflated. Acute endpoint of the ablation is considered a reduction of at least 25% of the HR or accelerated junctional rhythm.

### Redo procedures

All redo procedures were performed in general anaesthesia. The ablation strategy was the same for first redo and other redos. Anti-arrhythmic drugs and beta blockers were withheld for at least five half-lives prior to the procedure. A decapolar catheter was placed in the coronary sinus and a multipolar high density catheter was used to map the RA. Mapping and ablation were performed with one of the following: (1) PentaRay™ and THERMOCOOL SMARTTOUCH™ with CARTO mapping system (Biosense Webster, Diamond Bar, California), (2) Advisor™ HD Grid and TactiCath™ Contact Force Ablation Catheter, Sensor Enabled™ with EnSite Precision or EnSite X (St. Jude Medical, St. Paul, Minnesota), or (3) INTELLAMAP ORION™ and INTELLANAV STABLEPOINT™ with Rhythmia mapping system (Rhythmia Medical/Boston Scientific, Marlborough, Massachusetts). Ablation was performed using radiofrequency energy or pulsed field (PF) energy. CENTAURI PEF system (Galaxy Medical) or FARAPULSE™ Pulsed Field Ablation System (Boston Scientific, Marlborough, Massachusetts) were used for PF ablation.

A three-dimensional activation/voltage map was created using the appropriate catheter to each mapping system. A baseline electrophysiologic study was performed for differential diagnosis between IST recurrence vs. other supraventricular tachycardias. Bipolar activation mapping was used to identify and tag the earliest site (SN location) referenced to both an endocardial fiducial point (e.g. coronary sinus electrogram) and the surface *P*-wave. An assessment of block of SVC, crista terminalis, and IVC line was performed based on activation map and pacing manoeuvres. If the redo procedure was performed for recurrent IST and lines were not blocked, ablation was performed to obtain complete block of the lesion set and to reach the acute endpoint. If the line block was already confirmed at the beginning of the procedure or was not sufficient to reach the acute endpoint, additional applications on the crista terminalis were performed until the acute endpoint was achieved. Acute endpoint of the ablation was considered as a reduction of at least 25% of the HR or accelerated junctional rhythm. If the redo procedure was performed for other supraventricular tachycardias (e.g. atrial tachycardia or junctional tachycardia) ablation was performed based on the underlying electrophysiological mechanism. Inappropriate sinus node tachycardia lesion set was completed if the lines were not blocked. SN ablation and PM implantation was performed as last therapeutic option in symptomatic IST, refractory to SN sparing ablation, after a shared decision with the patient. All redo procedures were endocardial, and no redo epicardial ablation was performed.

### Follow-up

Patients were followed up in the outpatient clinic every 6 months and by remote monitoring for patients with cardiac implantable electronic devices (CIEDs). CIEDs patients underwent serial device interrogations every 6 months. Patients without CIEDs underwent 24 h Holter-ECG at 1 month, 3 months, 6 months, and every 6 months. The frequency domain measures of HR variability before and after hybrid ablation and before and after redo procedure were analysed, as previously described.^9,14^ The primary endpoint was first redo procedure. A redo procedure was deemed indicated if a symptomatic IST recurrence or symptomatic other supraventricular tachycardias occurred. The secondary endpoint was second or more redo procedures after first redo. A blanking period of 6 months after index hybrid procedure was taken into consideration as previously described.^9^ The primary safety endpoint was PM implantation after hybrid IST ablation. Pacemaker implantation was adjudicated as procedure related (sick sinus syndrome as a complication after hybrid IST ablation) or as a therapeutic choice in non-responder to hybrid SN sparing ablation. Secondary safety endpoint was complications after redo ablation. The following scores were evaluated baseline and 6 months after hybrid ablation, as previously described^12^: 1) assessment of quality of life (QoL) was performed with the SF-36 scoring system, 2) anxiety was assessed with Self-rating Anxiety Score (SAS), and 3) depression was evaluated with Zung Self-rating Depression Score (SDS).

### Statistical analysis

All variables were tested for normality with Shapiro–Wilk test. Normally distributed variables were described as mean ± standard deviation and the groups were compared through ANOVA, paired or unpaired *t*-test as appropriate, while the non-normally distributed variables were described as median (Inter Quartile Range) and compared by Mann–Whitney test or Wilcoxon signed-rank test as appropriate. The categorical variables were described as frequencies (percentages) and compared with Chi-squared test or Fisher’s exact test as appropriate.

Kaplan–Meier survival analysis was performed to analyse the cumulative event rates after blanking period (6 months). Survival analysis was performed with *survival* and *survminer* packages on R software.

A *P*-value less than 0.05 was considered statistically significant.

The analysis was performed using R software version 3.6.2 (R Foundation for Statistical Computing, Vienna, Austria) and SPSS Statistics 23.0 (IBM Corp, Armonk, New York, USA).

## Results

### Study population characteristics

A total of 223 patients with drug-resistant IST/POTS were screened and 220 patients undergone to hybrid IST ablation (98.6%) were included in the final analysis. Three patients (1.3%) refused hybrid IST ablation and opted for SN ablation with PM implantation and were excluded, *Figure 1*. The mean age was 31.0 years ± 11.7 years, and 195 patients (88.6%) were females. A total of 185 patients (84.1%) were treated for IST, and 61 patients (27.7%) were treated for POTS. An overlap phenotype was observed in 26 patients (11.8%).

Out of 220 patients treated with hybrid SN sparing IST ablation, 34 patients (15.4%) underwent to at least one redo procedure. The baseline characteristics of patients, stratified according to having or not a redo procedure, are summarised in *Table 1*. Compared to patients without any redo procedure, those undergoing at least one redo procedure demonstrated higher BMI [25.1 ± 4.8 vs. 23.4 ± 4.7, *P* = 0.039] and higher incidence of POTS [14 patients (41.2%) vs. 47 patients (25.2%), *P* = 0.045], *Table 1*.

**Table 1:** Baseline characteristics of the study population

Overall cohort	Total	No redo ablation	Redo ablation	*P*-value
	*N* = 220	*N* = 186	*N* = 34	
Age, years	31.0 ± 11.7	31.1 ± 11.0	30.9 ± 12.7	0.98
Female sex	195 (88.6%)	166 (89.2%)	29 (85.3%)	0.50
**BMI, kg/m^2^**	**24.1 ± 4.8**	**23.4 ± 4.7**	**25.1 ± 4.8**	**0**.**039**
LVEF > 55%	220 (100%)	186 (100%)	34 (100%)	NA
IST	185 (84.1%)	158 (84.9%)	27 (79.4%)	0.42
**POTS**	**61 (27.7%)**	**47 (25.2%)**	**14 (41.2%)**	**0**.**045**
Duration of symptoms, months	12.4 (4.2–22.5)	11.7 (4.2–21.5)	13.2 (3.8–31.0)	0.43
History of syncope	56 (25.4%)	47 (25.2%)	9 (26.5%)	0.88
Previous SP ablation	73 (33.2%)	59 (31.7%)	14 (41.2%)	0.28
Heart rate baseline, BPM	111.7 ± 5.6	111.4 ± 5.9	113.1 ± 7.4	0.9
Heart rate 24-h after procedure, BPM	66.5 ± 3.4	66.7 ± 3.9	65.5 ± 6.7	0.8
**Heart rate 6 months, BPM**	76.4 ± 5.4	73.7 ± 5.5	91.4 ± 8.7	<0.001
Heart rate 1 year, BPM	75.7 ± 5.6	75.6 ± 5.8	75.9 ± 7.7	0.9
**Medications**				
Ivabradine	214 (97.3%)	181 (97.3%)	33 (97.1%)	0.9
β-blockers	57 (25.9%)	48 (25.8%)	9 (26.5%)	0.9
Calcium channel blockers	212 (96.3%)	178 (95.7%)	34 (100%)	0.6
Class Ic antiarrhythmic drugs	42 (19.1%)	35 (18.8%)	7 (20.6%)	0.81

Redo cohort	Total	1 redo ablation	>1 redo ablation	*P* value
	*n* = 34	*n* = 16	*n* = 18	
Age, years	30.9 ± 12.7	30.7 ± 12.2	31.2 ± 13.4	0.89
Female sex	29 (85.3%)	13 (81.3%)	16 (88.9%)	0.64
BMI, kg/m^2^	25.1 ± 4.8	24.6 ± 6.6	25.4 ± 2.9	0.66
LVEF > 55%	34 (100%)	16 (100%)	18 (100%)	NA
IST	27 (79.4%)	11 (68.8%)	16 (88.9%)	0.21
POTS	14 (41.2%)	6 (37.5%)	8 (44.4%)	0.73
Duration of symptoms, months	13.2 (3.8–31.0)	16.0 (4.4–32.4)	13.2 (3.3–28.5)	0.64
History of Syncope	9 (26.5%)	5 (31.3%)	4 (22.2%)	0.70
Previous SP ablation	14 (41.2%)	7 (43.8%)	7 (38.9%)	1.00

Continuous variables are shown as mean ± standard deviation (SD) or median and (interquartile range) (IQR). Discrete variables are presented as numbers and percentages (%). In bold is significant comparisons.

Abbreviation list: BMI, body mass index; IST, inappropriate sinus tachycardia; LVEF, left ventricular ejection fraction; POTS, postural orthostatic tachycardia syndrome; SP, slow pathway.

Out of 34 patients with at least one redo procedure, 16/34 had only one redo and 18/34 had more than one redo procedures. There was no difference in the baseline characteristics between patients undergoing one vs. patients with more than one redo procedures, *Table 1*. The frequency domain measures of HR variability are summarised in Supplementary material online, *Table S1*. Self-rating Anxiety Score, SDS, and SF-36 QoL scores at baseline and 6 months after hybrid IST ablation are reported in Supplementary material online, *Table S2*.

### Follow-up

**Figure 2:**
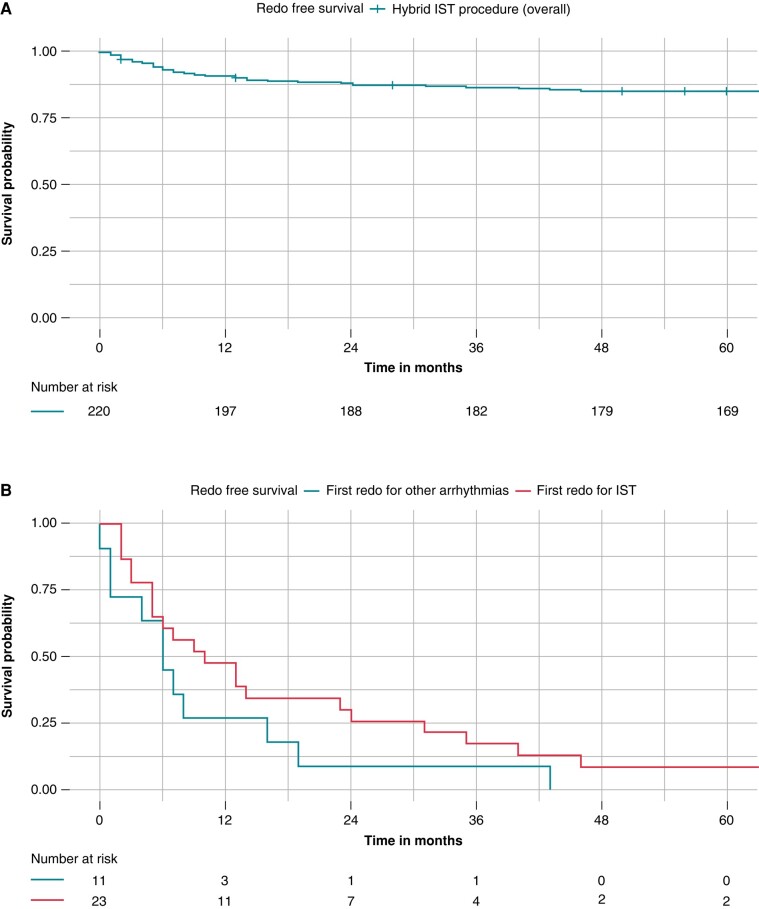
Kaplan–Meier curves of survival free from redo procedure after hybrid sinus node sparing ablation. Kaplan–Meier curves of survival free from redo procedures after hybrid inappropriate sinus node tachycardia (IST) ablation. Panel A, Kaplan–Meier curve of survival free from any redo procedure in the whole 220 patient cohort. After a mean follow-up of 73.3 ± 16.2 months, freedom from redo procedures was obtained in 84.6% of patients. Panel B, Patients with IST recurrence had the first redo at mean follow-up of 19.1 ± 20.8 months (blue curve), compared with patients with other arrhythmias, undergoing to a redo at a mean follow-up of 10.1 ± 12.4 months (red curve).

After a mean follow-up of 73.3 ± 16.2 months, 34 patients (15.4%) underwent a redo procedure after hybrid SN sparing ablation. A first redo procedure was performed at a mean follow-up of 16.2 ± 18.8 months, *Figure 2*. Out of 34 patients, a total of 23/34 patients had a redo procedure for IST recurrence and 11/34 patients for other arrhythmias. POTS patients accounted for 14/34 redo procedures but only one patient had a redo procedure for recurrent syncope. Patients with IST recurrence had the first redo at mean follow-up of 19.1 ± 20.8 months, compared with patients with other arrhythmias, undergoing to a redo at a mean follow-up of 10.1 ± 12.4 months, *Figure 2*. Out of 34 patients, 18/34 had a second redo. At second redo IST recurrence was diagnosed in 12/18 patients and other arrhythmias in 6/18 patients, *Table 2*. All 2 patients (5.9%) undergoing to a first redo procedure with FARAPULSE™ PFA had a second redo, while out of the four patients (11.8%) undergoing to a first redo with Galaxy PFA no patient had a second redo, *Table 2*.

**Table 2:** Procedural characteristics of redo procedures

	Total	1 redo ablation	>1 redo ablation^a^	*P*-value
	*n* = 34	*n* = 16 (47.0%)	*n* = 18 (53.0%)	
IST at redo procedure	23 (67.6%)	11 (68.8%)	12 (66.7%)	1.00
Blocked lines (SVC, IVC, CT)	4 (17.4%)	2 (18.2%)	2 (16.7%)	1.00
Ablation on CT	18 (78.3%)	9 (81.8%)	9 (75.0%)	1.00
Ablation on SVC	4 (17.4%)	2 (18.2%)	2 (16.7%)	1.00
Ablation on IVC	0 (0.0%)	0 (0.0%)	0 (0.0%)	NA
PF ablation				
FARAPULSE**™**	2 (8.7%)	0 (0.0%)	2 (15.4%)	0.48
**Galaxy Medical**	**4 (17.4%)**	**4 (36.4%)**	**0 (0.0%)**	**0**.**03**
RF ablation	17 (73.9%)	7 (63.6%)	10 (83.3%)	0.37
Others arrhythmias at redo procedure	11 (32.4%)	5 (31.3%)	6 (33.3%)	1.00
AT right sided	5 (45.5%)	1 (20.0%)	4 (66.7%)	0.24
AFL typical	3 (27.3%)	2 (40.0%)	1 (16.7%)	0.54
JT	2 (18.2%)	2 (40.0%)	0 (0.0%)	0.18
AF	1 (9.1%)	0 (0.0%)	1 (16.7%)	1.00
Blocked lines (SVC, IVC, CT)	2 (22.2%)	1 (20.0%)	1 (25.0%)	1.00
Ablation on CT	6 (66.7%)	3 (60.0%)	3 (75.0%)	1.00
Ablation on SVC	1 (11.1%)	1 (20.0%)	0 (0.0%)	1.00
Ablation on IVC	0 (0.0%)	0 (0.0%)	0 (0.0%)	NA
PF (Galaxy Medical) ablation	1 (9.1%)	0 (0.0%)	1 (16.7%)	1.00
RF ablation	10 (90.9%)	5 (100%)	5 (83.3%)	1.00

Continuous variables are shown as mean ± standard deviation (SD). Discrete variables are presented as numbers and percentages (%). In bold is significant comparisons.

Abbreviation list: AF, atrial fibrillation; AFL, atrial flutter; AT, atrial tachycardia; CT, crista terminalis; IST, inappropriate sinus tachycardia; IVC, inferior vena cava; JT, junctional tachycardia; PF, pulse field; RF, radiofrequency; SVC, superior vena cava.

^a^In the cohort of more than one redo, the data refer to the second redo.

### Redo procedural data

**Figure 3:**
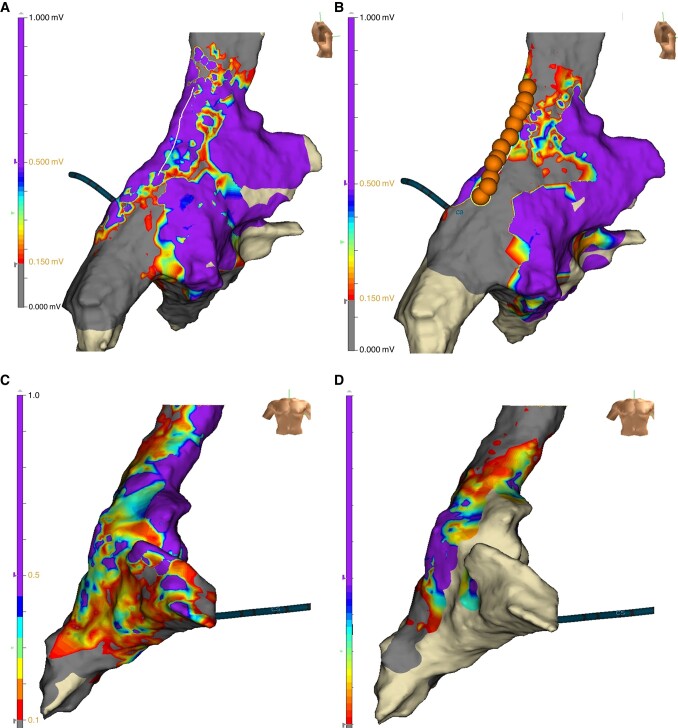
Electrophysiological findings at redo procedures after hybrid ablation. Endocardial mapping and ablation at redo procedures after hybrid inappropriate sinus node tachycardia (IST) ablation. Panel A and B, Patient with IST recurrence. Panel A, Endocardial voltage map performed with Advisor™ HD Grid ablation showed incomplete crista terminalis line. Ablation was performed with TactiCath™ Ablation Catheter, connected with CENTAURI pulsed field system (Galaxy Medical). Panel B, Remap after ablation showed block of the crista terminalis (ablation tags in orange). Panel C and D, Patient with IST recurrence. Panel C: Endocardial voltage map performed with Advisor™ HD Grid ablation showed incomplete superior vena cava isolation. Ablation was performed with TactiCath™ Ablation Catheter, connected with CENTAURI pulsed field system (Galaxy Medical). Panel D: Remap after ablation showed superior vena cava isolation.

In the group of 23 patients (67.6%) with IST recurrence, 19/23 patients had at least one line (SVC, IVC or CT) not blocked and 18/23 patients had a gap in the CT line requiring an endocardial ablation, *Figure 3*. Four patients had lines blocked and additional applications were performed on CT line. Radiofrequency ablation was performed in 17 patients (73.9%) and PF ablation in six patients (26.1%). All lines were blocked at the end of the procedure, and the acute endpoint was reached in all patients. Eleven patients (32.4%) had a redo for other arrhythmias different from IST as follows: five patients (14.7%) for a right atrial tachycardia (scar related and dependent on not-blocked CT line), three patients (8.8%) for typical atrial flutter, two patients (5.9%) for junctional tachycardia, and one patient (2.9%) for atrial fibrillation, *Table 2*. Of these patients, seven patients (77.7%) had at least one not-blocked line (SVC, IVC, or CT), and six patients (66.7%) had a gap in the CT line requiring an endocardial ablation in this site. Radiofrequency ablation was used in 10 patients (90.9%) and PF ablation in one patient (9.1%). Ablation was performed with success in all patients, based on the arrhythmia diagnosed and all lines were blocked, if a gap was found.

Procedural data stratified according to one or more than one redo procedure are reported in *Table 2*.

### Safety endpoints

**Figure 4:**
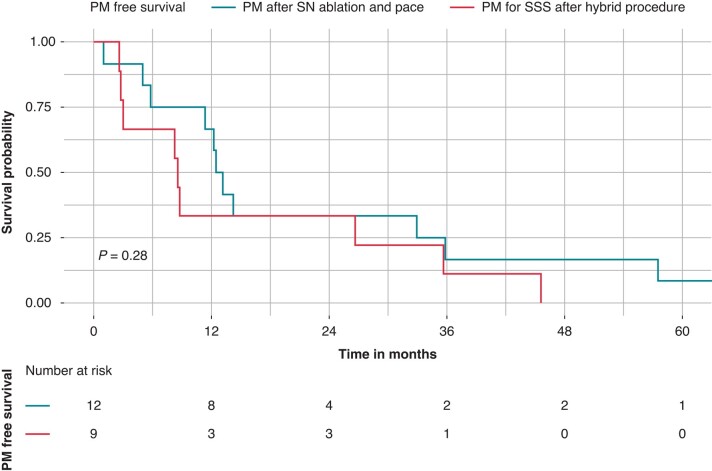
Kaplan–Meier curves of survival free from pacemaker implantation after hybrid sinus node sparing ablation. Kaplan–Meier curves of survival free from pacemaker (PM) implantation after hybrid inappropriate sinus node tachycardia (IST) ablation. Nine patients (4.1%) did not undergo a redo procedure and experienced a sick sinus syndrome requiring dual-chamber PM implantation after a mean follow-up of 15.7 ± 16.1 months (red curve). A total of 12 patients (5.4%) were implanted after a mean of 22.8 ± 22.4 months with a dual-chamber PM as a shared therapeutic choice combined with sinus node (SN) ablation procedure.

Over the follow-up period, 21 patients (9.5%) underwent to a PM implantation, *Figure 4*. Of these, nine patients (4.1%) did not undergo a redo procedure and experienced a sick sinus syndrome requiring dual-chamber PM implantation after a mean follow-up of 15.7 ± 16.1 months. The remaining 12 patients (5.4%) were implanted after a mean of 22.8 ± 22.4 months with a dual-chamber PM as a shared therapeutic choice combined with SN ablation procedure. The rate of therapeutic PM implantation was significantly higher in patients undergoing a redo procedure compared to those without a redo procedure [12 patients (35.3%) vs. nine patients (4.8%), *P* < 0.001]; however there was no difference between patients undergoing more than one redo procedure compared to those undergoing one redo procedure [nine patients (50.0%) vs. three patients (18.8%), *P* = 0.08], *Table 3*. Complete characteristics of patients with PM implantation for sick sinus syndrome post hybrid IST ablation are summarised in *Table 4*. One patient (2.9%) experienced a complication after a redo procedure. In particular, in this patient with POTS and recurrent debilitating refractory episodes of syncope, a redo procedure for neuromodulation was performed with ablation of the right pulmonary vein ganglionated plexus. The patient developed gastroparesis that required a gastrostomy.

**Table 3:** Pacemaker implantation in the study population

	Total	No redo IST	Redo IST	*P*-value
	*n* = 220	*n* = 186	*n* = 34	
**PM**	**21 (9.5%)**	**9 (4.8%)**	**12 (35.3%)**	**< 0.001**
SSS (no redo)	9 (4.1%)	9 (4.8%)	0 (0.0%)	NA
SN ablation/mod (1 redo)	12 (5.4%)	0 (0.0%)	12 (35.3%)	NA

	Total	1 redo IST	1+ redo IST	*P* value
	*N* = 34	*N* = 16 (47.0%)	*N* = 18 (53.0%)	
PM	12 (35.3%)	3 (18.8%)	9 (50.0%)	0.08

Continuous variables are shown as mean ± standard deviation (SD) or median and interquartile range (IQR). Discrete variables are presented as numbers and percentages (%). In bold is significant comparisons.

Abbreviation list: IST, inappropriate sinus tachycardia; PM, pacemaker; SN, sinus node; SSS, sinus node sick syndrome.

**Table 4:** Pacemaker implantation for sick sinus syndrome post hybrid procedure

Patient	Sex	Age	BMI	Months from first procedure to PM	Comorbidities
#1	Female	36.2	22.5	35.6	SLE
#2	Female	21.0	22.4	2.9	/
#3	Female	45.6	25.9	2.5	/
#4	Female	50.8	17.6	8.7	AF
#5	Female	21.7	16.8	8.2	EDS
#6	Female	22.3	19.6	26.6	/
#7	Female	20.3	23.1	45.6	/
#8	Female	53.9	29.4	8.5	AF
#9	Male	36.4	22.4	2.7	/

Abbreviation list: AF, atrial fibrillation; BMI, body mass index; EDS, Ehlers-Danlos syndrome; SLE, systemic lupus erythematosus.

## Discussion

The main findings of the current study are the following: 1) at long-term follow-up of ≍6 years, 15.4% of patients after hybrid IST ablation underwent to a redo procedure; 2) in most patients undergoing a redo procedure, a gap in at least one previous line was found and the most common gap was in the CT line; 3) PM implantation for sinus node sick syndrome (SSS) after hybrid ablation was low (4.1%) and consistent with previous data.

### Hybrid sinus node sparing ablation outcomes

Previous studies documented the efficacy and safety of the novel hybrid endocardial/epicardial SN sparing approach in drug-resistant IST/POTS.^9,12,14^ Based on these promising results the HEAL-IST trial (NCT05280093), an international, multicentre, investigational device exempt (IDE) trial on hybrid SN sparing ablation is ongoing.^15^

The current study is the first to analyse redo procedures after hybrid IST ablation. At long term follow-up of ≍6 years, 15.4% of patients underwent a subsequent redo ablation. In the previous SUSRUTA-IST registry, at 1 year follow-up, 8% of patients underwent a redo procedure after hybrid SN sparing ablation vs. all (100%) of patients in the standard SN modification ablation group.^12^

Pacemaker implantation rate for SSS after hybrid IST ablation in this study was as low as 4.1% and compatible with previous literature. The presented results expand on a larger cohort and with longer follow-up our previous data from SUSRUTA-IST registry reporting a PM implantation rate of 4% after hybrid IST ablation vs. 50% in the standard SN modification ablation group.^12^ The development of SSS is likely attributed to the epicardial lesions performed next to SN area, despite localising the SN through the endocardial mapping. However, when analysing the data of these nine patients, only two of them (Patients 6 and 7 in *Table 4*) are young women without any alternative cause that could explain the occurrence of SSS. Interestingly, Patients 6 and 7 developed SSS after more than 2 years from the hybrid procedure, at 26.6 and 45.6 months, respectively. Patient 2, despite being young and with no comorbidities, underwent a RF endocardial ablation next to SN area during hybrid ablation. The other six patients were older patients, and the co-existence of a ‘sick atrium’ due to a longer history of IST may be related to an early onset of SSS, as previously described.^16^ Fibrocytes and active fibroblasts were found in the histological and ultrastructural analyses of the SN excised from IST patients indicating extracellular fibrosis.^17^

### Redo procedures after hybrid sinus node sparing ablation

In most patients (67.6%), a redo procedure was performed for IST recurrence. In patients with IST recurrence, 82.6% had at least one not-blocked epicardial line, and 78.3% had a gap in the CT line requiring an endocardial ablation in this site. This might be explained by technical challenges in positioning of the clamp over the right atrium along the crista terminalis. Indeed, a higher mean BMI is found in the redo cohort compared to patients without a redo. The presence of abdominal visceral fat and the resulting elevation of the right hemidiaphragm can hinder the surgeon’s full access to anatomical structures, in particular the lower portion of the CT line. In this case endocardial mapping and ablation at first hybrid procedure is of utmost clinical importance.^18^

In patients with a recurrence for an arrhythmia different from IST, ≍80% of patients had at least one not-blocked line and five patients had a scar related right atrial tachycardia dependent on not-blocked CT line.

Compared with CT line, IVC line was always found blocked, and a low rate of incomplete SVC isolation was observed (17.4% in IST group and 11.1% in no IST group). This may be related to a better performance of epicardial ablation in these sites compared to CT line. However, besides technical challenges, from an anatomical standpoint the CT, is a thick muscular ridge with a length of 40–55 mm and a thickness of > 7 mm.^19^ The tissue thickness is a major factor for transmurality after ablation, especially considering the different energy sources currently available.^20^

Radiofrequency was the most used energy source for redo procedures; however, seven patients underwent PF ablation. In two patients FARAPULSE™ Pulsed Field Ablation System (Boston Scientific, Inc.) was used to complete CT line, and both patients underwent a subsequent redo procedure with evidence of not blocked CT line (reconnection).^21^ On the other hand, the use of Galaxy Medical CENTAURI System to close the gap on CT was not associated with any further redo procedure or any recurrence. This system has been demonstrated to deliver lesions up to 8.2 mm in depth, providing a rationale for its clinical efficacy in IST redo ablation.^22,23^

### Limitations

The current study is a retrospective single centre study. One experienced surgeon (MLM) performed all the cases (epicardial ablation). Different electrophysiologists performed the endocardial procedure (both hybrid and redo procedures). The reproducibility of the approach might be reduced; however, this will be tested in the international, multicentre HEAL-IST trial (NCT05280093).^15^ Limitations include referral bias due to the inclusion of patients from a tertiary centre patients specialised in hybrid ablation. Our centre is a European referral centre for IST treatment. Patients are referred both after diagnosis and after failure of pharmacological and non-pharmacological therapy (by primary care physicians, cardiologists, and neurologists). For this reason, to provide an estimate of the general population out of which the procedures are performed, might be biased. Reporting patients not qualifying for the hybrid ablation (e.g. patients with IST and a good response to pharmacological or non-pharmacological therapy) is outside the scope of the current study. Although the PM implantation rate for SSS is low, the development of a better way to identify/protect SN area could reduce the incidence of SSS and PM implantation in this population.

## Conclusions

In a large cohort of patients undergoing to hybrid SN sparing IST ablation, the long-term free survival from redo procedure was 84.6%. A total 15.4% of patients underwent to a redo procedure and in most a gap in the CT line was found. Pacemaker implantation for SSS after hybrid ablation was low (4.1%) and consistent with previous data.

## Supplementary Material

euad373_Supplementary_Data
