# Supplementary material for: Redo procedures after sinus node sparing hybrid ablation for inappropriate sinus tachycardia/postural orthostatic sinus tachycardia
Source: Europace. 2023 Dec 29;26(1):euad373. doi: 10.1093/europace/euad373 (PMC10775684; doi:10.1093/europace/euad373)
Supplement: euad373_Supplementary_Data [file euad373_supplementary_data.docx]

# Supplemental material

# Redo Procedures after Sinus Node Sparing Hybrid ablation for Inappropriate Sinus Tachycardia / Postural Orthostatic Sinus Tachycardia

# Supplemental Tables

## Supplemental Table 1. Time-domain heart rate variability.

| **Parameter** | **Definition** | **Pre-Hybrid ablation (N=220)** | **Post-Hybrid ablation (N=220)** | **Pre-redo ablation (N=34)** | **Post-redo ablation (N=34)** |
| --- | --- | --- | --- | --- | --- |
| **Mean NN (ms)** | **Mean of all normal RR intervals (normal to normal coupling interval)** | 545.6 ± 29.7 | 1032.8 ± 158.8 | 548.4 ± 46.9 | 1040.9 ± 177.5 |
| **SDNN (ms)** | **Standard deviation of all normal RR intervals (SDRR or CLV)** | 98.7 ± 13.2 | 145.6 ± 46.4 | 99.5 ± 15.3 | 148.9 ± 49.8 |
| **SDANN** | **Standard deviation of mean RR interval for all 5-minute segments of 24-hour ECG recordings** | 71.7 ± 20.8 | 127.5 ± 31.4 | 74.3 ± 22.1 | 129.3 ± 35.8 |
| **SD (ms)** | **Mean of standard deviations of all normal RR intervals for all 5-min segments**  **of a 24-h ECG recording** | 24.2 ± 3.8 | 64.3 ± 12.4 | 27.4 ± 5.9 | 68.4 ± 15.7 |
| **rMSSD (ms)** | **Root mean square successive differences between adjacent normal RR intervals**  **over the entire 24-h ECG recordings** | 17.5 ± 8.3 | 37.8 ± 9.2 | 18.7 ± 9.5 | 38.4 ± 12.3 |
| **pNN50 (%)** | **Percent of difference between adjacent normal RR intervals that are greater than 50 ms computed over the entire 24-h ECG recordings** | 4.3 ± 3.8 | 16.0 ± 7.4 | 4.4 ± 4.0 | 17.0 ± 8.2 |

## Supplemental Table 2. SAS, SDS, SF-36 QoL scores baseline and 6 months after hybrid IST ablation.

| **Score** | **Pre-Hybrid ablation (N=220)** | **6-months Post-Hybrid ablation (N=220)** | **p value** |
| --- | --- | --- | --- |
| **SDS (Depression)** | 33.0 (28.0-36.0) | 25.0 (23.0-30.0) | <0.001 |
| **SAS (Anxiety)** | 35.0 (33.5-36.0) | 24.0 (22.0-29.0) | <0.001 |
| **SF-36 Functional Domains** |  |  |  |
| **Physical Functioning** | 84.0 (81.0-93.0) | 93.0 (87.0-98.0) | <0.001 |
| **Role Physical** | 84.0 (76.0-100.0) | 100.0 (89.5-100.0) | <0.001 |
| **Bodily Pain** | 100.0 (69.0-100.0) | 92.0 (90.0-100.0) | 0.56 |
| **General Health** | 64.5 (51.5-76.5) | 76.0 (66.5-88.5) | <0.001 |
| **Vitality** | 85.0 (70.0-89.0) | 93.0 (81.0-97.0) | <0.001 |
| **Social Functioning** | 84.0 (74.0-100.0) | 100.0 (91.0-100.0) | <0.001 |
| **Role Emotional** | 62.0 (60.0-74.0) | 77.0 (62.0-82.0) | <0.001 |
| **Mental Health** | 66.5 (64.5-82.5) | 81.0 (75.0-87.0) | <0.001 |
